# Challenges and opportunities in newly diagnosed glioblastoma in the United Kingdom: A Delphi panel

**DOI:** 10.1093/nop/npae058

**Published:** 2024-06-22

**Authors:** Helen Bulbeck, Karen Noble, Kathy Oliver, Tina Mitchell Skinner

**Affiliations:** Brainstrust—The Brain Cancer People, Cowes, UK; Brain Tumour Research, Milton Keynes, UK; The International Brain Tumour Alliance (IBTA), Tadworth, UK; Brain Tumour Support, Thornbury, UK

**Keywords:** access to clinical trials, Delphi panel, newly diagnosed glioblastoma, UK, unmet need

## Abstract

**Background:**

Glioblastoma is the deadliest primary malignant brain tumor in adults with limited treatment options and an average survival time of 12–18 months in the United Kingdom. In addition, glioblastoma has a highly detrimental impact on physical, cognitive, and emotional well-being, leaving substantial unmet needs for patients and caregivers. This study aimed to identify unmet needs in people with newly diagnosed glioblastoma and opportunities to mitigate them.

**Methods:**

Utilizing Delphi methodology, an initial roundtable discussion with patient advocacy experts from 5 brain tumor organizations in the United Kingdom informed the development of 2 rounds of surveys across 9 domains (diagnosis, treatment, integrated care, support beyond treatment, quality of life, access to new treatments, access to trials, measures to ease the burden, and impact of COVID-19). Consensus was predefined as ≥70% agreement.

**Results:**

A total of 17 Delphi panelists (glioblastoma patients, caregivers, and patient representatives) completed the first round of questionnaires and 26 completed the second. Consensus was reached on 16/21 questions (76.2%) and 7/9 domains. Panelists reached a consensus on key questions including the high frequency of diagnosis via emergency departments, the lack of effective personalized treatments and holistic care, the high caregiver burden, the lack of awareness and availability of access to clinical trials, and the negative impact of COVID-19 on glioblastoma care.

**Conclusions:**

Significant unmet needs exist for newly diagnosed glioblastoma patients in the United Kingdom, highlighting the demand for increased research funding, comprehensive patient care, caregiver support, enhanced awareness and access to clinical trials, and new treatments.

Glioblastoma (GBM) is the most common and aggressive primary malignant brain tumor.^1,2^ GBMs are grade 4 tumors as categorized by the World Health Organization classification system and are associated with a poor prognosis.^3^ Despite current treatment options, consisting of surgery, radiotherapy and temozolomide, the average survival time for GBM patients is 12–18 months, with approximately 25% surviving beyond 1 year and only 5% surviving longer than 5 years.^1^ This intransigent disease has a significant impact on a person’s quality of life as it is a highly malignant type of cancer that attacks an individual’s physical, cognitive and emotional well-being.^4^

The standard first-line treatment for newly diagnosed GBM in the United Kingdom is maximal safe surgical resection (if appropriate) followed by concurrent chemoradiotherapy. The concurrent chemoradiotherapy consists of continuous oral temozolomide chemotherapy administered daily (including weekends for the full duration of the radiotherapy) along with fractionated radiotherapy totaling 60 gray (gy) administered in 30 fractions over 6 weeks during weekdays.^5^ However, other fractionation schedules, such as 40 Gy in 15 fractions, are also supported by randomized data and may be offered to older patients as another acceptable standard.^6^ Following this, patients continue with maintenance temozolomide chemotherapy, consisting of chemotherapy for 5 days every 28-day cycle, for up to 6 cycles if they have had maximal safe surgical resection or a biopsy, or up to 12 cycles if necessary.^5,7^ However, even with optimal first-line treatment, prognosis remains poor. Despite treatment, nearly all GBMs recur, at which point there is no standard protocol and therapies are limited to re-resection (if appropriate), re-irradiation (if appropriate) and/or systemic therapies upon tumor progression.^5^

In the United Kingdom, there are an estimated 3200 new cases of GBM diagnosed each year.^8^ Given the aggressive nature and poor prognosis of GBM, newly diagnosed patients and their caregivers face immense physical, psychosocial and emotional challenges.^9^ However, there is a limited amount of research which explores the patient perspective and lived experience with newly diagnosed GBM specifically in the UK context. Engaging patients, caregivers, and patient advocates is crucial to reaching an expert consensus on the key issues that affect the GBM patient journey and potential opportunities to mitigate them.

The Delphi panel methodology follows a scientific method for achieving expert consensus on specific issues utilizing a series of surveys and controlled feedback until consensus is established and summarized. Across the literature, consensus is usually considered to be reached when the agreement is between 55% and 100%.^10^ Delphi panels can be used to elucidate the opinion of a disease community on highly burdensome illnesses for which there are high unmet needs in care, such as GBM.

This study aimed to identify key unmet needs, challenges, and priorities of newly diagnosed GBM patients and their caregivers in the United Kingdom, to explore potential strategies to address these issues and inform efforts to optimize care and support, by engaging patients, caregivers, and patients advocates in a comprehensive Delphi panel.

## Materials and Methods

### Study Design

This study utilized Delphi panel methodology, preceded by a roundtable discussion with the coauthors of this study to inform the subsequent development of a first-round questionnaire. As per the Delphi methodology, the first-round questionnaire consisted of a qualitative survey based on open-ended questions and was distributed to Delphi panelists. Responses were analyzed and questions were refined, leading to the creation of the second-round questionnaires consisting of quantitative and multiple-choice questions. The second-round questionnaire was shared again with the panelists.^11^

### Questionnaire Development

Prior to the Delphi panel, a roundtable discussion was conducted, in November 2022, with representatives from 5 UK-based brain tumor organizations, 4 of which are patient advocacy groups (PAGs), specializing in GBM and other brain tumors: the International Brain Tumour Alliance (IBTA), brainstrust—the brain cancer people, Brain Tumour Support, Brain Tumour Research, and the Tessa Jowell Brain Cancer Mission. The 90-min discussion provided insights from these organizations on the challenges and unmet needs of people with newly diagnosed GBM in the United Kingdom while exploring opportunities to help ease the disease burden on people with newly diagnosed GBM and their caregivers. A total of 9 domains were identified:

1. Diagnosis of GBM in the United Kingdom.
2. National Health Service (NHS) patient and treatment pathways.
3. Integrated care and multidisciplinary teams (MDTs).
4. Support beyond treatment.
5. Patient and caregiver quality of life.
6. Measures to ease the burden of GBM.
7. Access to new treatments on the NHS.
8. Access to clinical trials.
9. Impact of COVID-19 on GBM care.

Insights from the discussion informed the development of the first round questionnaire. The first round questionnaire was composed of 5 demographic questions to help characterize the population sample and 24 open-ended research questions on the 9 key domains identified in the roundtable, allowing Delphi panelists to brainstorm on each topic (see Supplementary Section A). All questions were reformulated in the second-round questionnaire and it was composed of closed questions based on the responses to the first-round questionnaire (see Supplementary Section B). As consensus was reached on most domains in the second round, it was decided that a third-round questionnaire would not be necessary, and the Delphi panel was concluded after the second round.

### Participant Recruitment

All recruited panelists were contacts of the 4 participating PAGs, as these groups are more likely to include people with lived experience of GBM. A Delphi panel facilitator drafted a template invitation to participate and follow-up emails for each questionnaire, and PAG representatives distributed the first and second round questionnaires through their mailing lists and newsletters. Recipients of the invitation emails and questionnaires included patients with a brain tumor (including GBM), current and former caregivers to someone living with a brain tumor (including GBM), and other PAG contacts. Participants completed both questionnaires anonymously and online via Microsoft Forms. Responses were collected from 30 January 2023 to 24 April 2023.

### Data Analysis

Microsoft Office Excel v16.74 was used for the statistical analysis of the Delphi data. All answers were reviewed and validated by 2 reviewers. For closed-ended questions in the second-round questionnaire, the percentage of participants selecting each option was calculated to determine consensus (see Section Consensus Determination). The second-round questionnaire included Likert-type items for participants to rate (6 levels) and, for determining consensus, the obtained responses were combined for the 2 highest options denoting agreement (eg, “strongly agree” and “agree”). Qualitative comments from the first round questionnaire were analyzed to complement the obtained quantitative responses. In both rounds, responses from participants who were not associated with GBM (those people diagnosed with or caring for someone with other types of brain tumors) were excluded. In the first round, 3 responses were excluded (2 patients and 1 caregiver), and in the second round, 2 responses from caregivers were excluded. After the exclusion of responses associated with types of brain tumors that were not GBM, 17 and 26 participants were included in the first and second rounds, respectively. Partially complete responses from eligible participants, who had skipped at least 1 or more questions, were analyzed for only the completed questions. In the first round, 7 out of the 17 included questionnaire responses which were partially completed, and in the second round, 12 out of the 26 included questionnaire responses which were partially completed.

### Consensus Determination

Consensus was defined as ≥70% agreement/strong agreement among respondents. This consensus threshold has been utilized in previous oncology Delphi studies.^12–14^ Open-ended questions from the first round was re-formulated in the second round as closed-ended questions for re-evaluation.

### Anonymity and Continuity of Respondents

To preserve anonymity, participants completed each round of questionnaires on Microsoft Forms, which allowed the third-party facilitator to set up the questionnaires to ensure that personally identifiable information was not collected from the participants. As such, it was not possible to ensure the same respondents participated across rounds, although the PAG representatives distributed each questionnaire via the same channels. To counter that, respondents were asked to self-report whether they had completed the previous round.

## Results

Consensus (defined as ≥70% agreement) was reached on a total of 16 of the 21 questions (76.2%) after the second round across 7 out of 9 domains.

### Participant Demographics

The Delphi study included 17 participants in the first round and 26 participants in the second round. Participants were either GBM patients or caregivers, with a mix of ages and geographic locations across the United Kingdom. In the second round, 62% (*n* = 16/26) of participants reported having also responded to the first-round questionnaire. Detailed participant demographics are presented in Supplementary Table 1.

### Diagnosis of Glioblastoma in the United Kingdom

All 26 participants in the second round responded to the question regarding the most common routes to a GBM diagnosis. A total of 21 (81%) participants agreed that a visit to an accident and emergency (A&E) department at a hospital was the most common route to a GBM diagnosis. As reported in the first round, A&E visits were often sought after symptoms had worsened or other causes had been ruled out. Three participants in the first round reported misdiagnosis by their general practitioners, who attributed their symptoms to migraines and failed to ask the patients further questions.

The main challenges associated with receiving a GBM diagnosis were identified as emotional and psychological burdens, with the majority of participants (*n* = 22/26, 85%) agreeing that there are challenges such as dealing with shock, lack of hope, fear of mortality and uncertainty about the future. Participants (*n* = 22/26, 85%) also agreed that there is a lot of information to take in about the disease and lifestyle changes following diagnosis. Some qualitative comments from the first-round survey results provided further insights into these emotional obstacles participants faced following a GBM diagnosis. They reported experiencing “feelings of doom and hopelessness according to the dire prognosis of survival average of 14 months even with ‘gold standard’ treatment” and “concern or desperation that even this time will be spent with time-consuming treatment with significant side effects.” Some other challenges reported in the first round were “first symptoms resulted in an emergency surgery to save a life,” “emergency ambulance to A&E following a seizure and being found unconscious” and “random seizure, hospital, admission, biopsy diagnosis within 2 weeks.”

**Figure 1.**
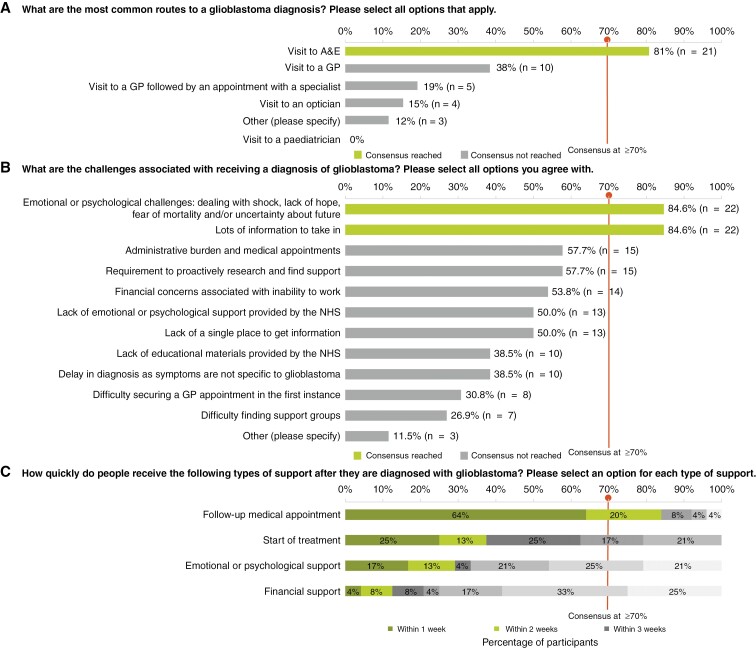
Diagnosis of glioblastoma in the United Kingdom. Participant responses rank (a) the most common routes to a glioblastoma diagnosis (b) the challenges associated with receiving a glioblastoma diagnosis and (c) reporting time span within which people received support following diagnosis. The consensus was defined as ≥70% agreement between participants, and responses subdivided into 3 sets: within 1 week; within 2 weeks; and within 3 weeks. Note: All 26 participants responded to questions (a) and (b). While 25 participants responded to question (c), 1 of them skipped some elements as shown in panel (c). Abbreviations: GP (general practitioner); NHS (National Health Service).

Participants (*n* = 21/25, 84%) agreed that they received a follow-up medical appointment within 2 weeks of a GBM diagnosis, and that treatment was initiated within 4 weeks of diagnosis (*n* = 19/24, 79%). The results for this domain are visualized in Figure 1.

### NHS Patient Pathways

Participants reached a consensus that the knowledge/expertise (*n* = 22/25, 92%) and quality of care received from specialist clinicians in the United Kingdom (*n* = 23/26, 89%) is “very good” or “good.” However, participants also identified substantial challenges and unmet needs that are “very relevant” or “relevant,” including the need for a more holistic approach to care (*n* = 23/26, 88%), lack of effective treatment options (*n* = 22/26, 85%), treatment options not having progressed for decades (*n* = 22/26, 85%), poor communication between healthcare professionals (*n* = 20/26, 77%), delayed or inadequate access to care and treatment (*n* = 19/26, 73%) and lack of support if treatment fails (*n* = 19/25, 76%).

**Figure 2.**
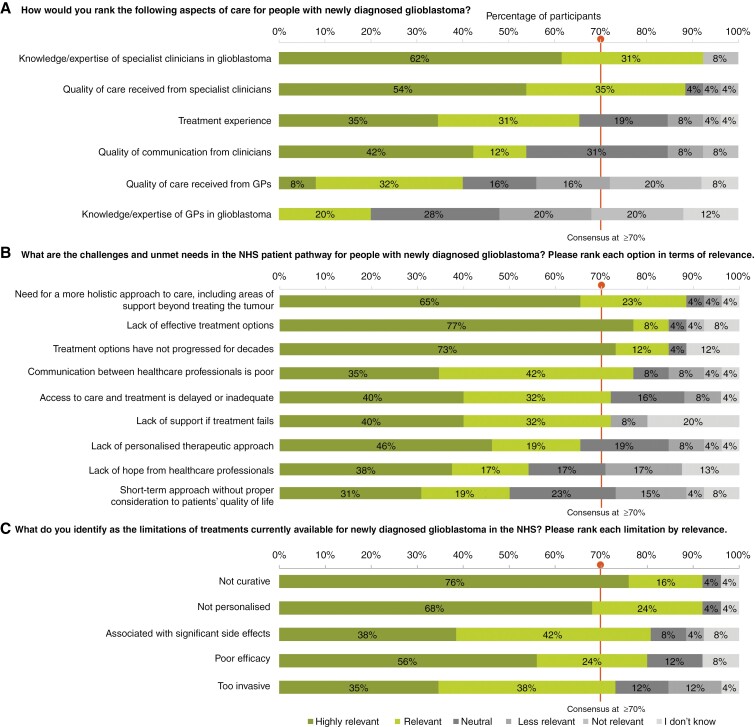
Participant responses ranking, in the context of newly diagnosed glioblastoma, (a) aspects of care; (b) challenges and unmet needs within the NHS patient pathway; (c) limitations of current treatments available via the NHS. Consensus was defined as ≥70% agreement between participants. Responses were ranked as “Highly relevant”; “Relevant”; “Neutral”; “Less relevant”; “Not relevant”; “I don’t know.” Note: All 26 participants responded to all 3 questions but 1 skipped part of the question shown in panel (a), 2 skipped parts of the question shown in panel (b) and 1 skipped part of the question shown in panel (c). Abbreviations: GP (general practitioner); NHS (National Health Service).

Participants also reached a consensus on the limitations of treatments currently available for newly diagnosed GBM in the NHS. These treatments are not curative, lack personalization, are associated with significant side effects, provide poor efficacy, and are overly invasive. The results for this domain are visualized in Figure 2.

### Integrated Care and Multidisciplinary Teams

**Figure 3.**
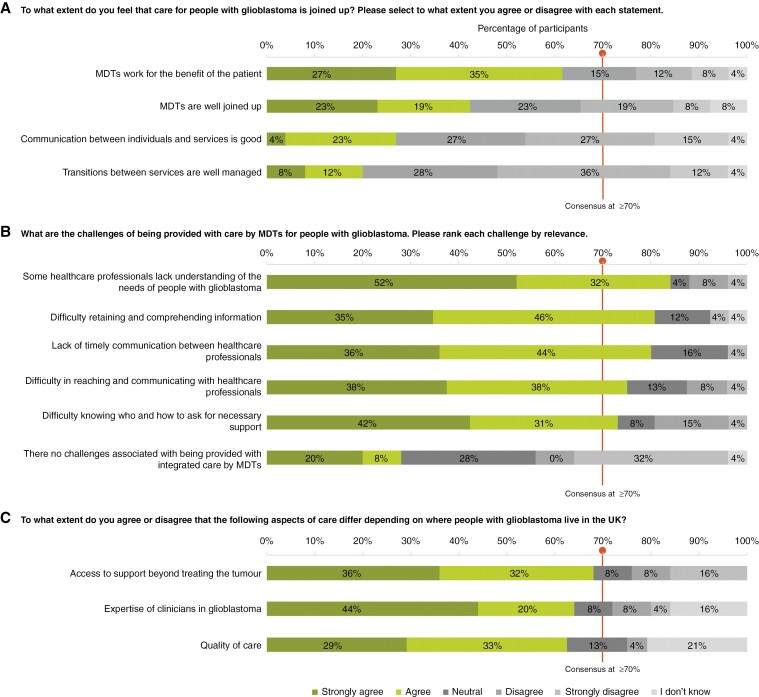
Participant responses show levels of agreement on (a) to what extent glioblastoma care is coordinated in the United Kingdom, (b) challenges in MDT patient care and (c) aspects of care patients/caregivers believe to differ by location within the United Kingdom. Consensus was defined as ≥70% agreement between participants. Responses were ranked according to the level of agreement as follows: “Strongly agree”; “Agree”; “Neutral”; “Disagree”; “Strongly disagree”; “I don’t know.” Note: All 26 participants responded to questions (a) and (b) and 25 to questions (c). One participant skipped part of the question shown in panel (a), 2 skipped parts of the question shown in panel (b) and 1 skipped parts of the question shown in panel (c). Abbreviations: MDT (multidisciplinary team); UK (United Kingdom).

Participants did not reach a consensus on the extent to which they believed that care for people with GBM is coordinated or on whether aspects of care differ depending on the location of GBM patients in the United Kingdom. However, 5 common challenges related to care provided by multidisciplinary teams (MDTs) were identified. These challenges include some healthcare professionals lacking an understanding of patient needs (*n* = 21/25, 84%), difficulty retaining details as there is a lot of information which the patient and caregiver have to on-board and process (*n* = 21/26, 81%), poor communication between healthcare professionals (*n* = 20/25, 80%), difficulty contacting professionals (*n* = 18/24, 75%), and difficulty knowing who or how to ask for support (*n* = 19/26, 73%). One of the comments also reported was that the caregiver “often feels left out of the process of care decisions.” The results for this domain are visualized in Figure 3.

### Support Beyond Treatment

**Figure 4.**
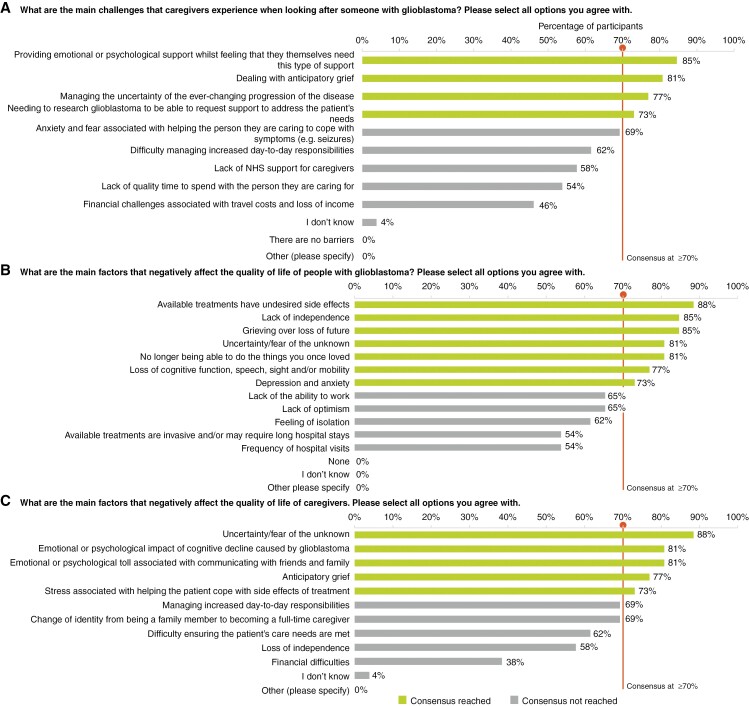
Participant responses showed levels of agreement on (a) the main challenges faced by caregivers, (b) factors that negatively affect the quality of life of glioblastoma patients, and (c) their caregivers. Consensus was defined as ≥70% agreement between participants. Note: All 26 participants responded to all questions and no parts of any questions were skipped. Abbreviation: NHS (National Health Service).

Participants did not reach consensus on the types of support, beyond treatment, provided to people with newly diagnosed GBM within the NHS. In the first round of responses, multiple PAGs were mentioned by participants as providing helpful support in various capacities, including The Brain Tumour Charity, brainstrust—the brain cancer people, Macmillan Cancer Support, Marie Curie, and Brain Tumour Support. The types of support described included information booklets, financial assistance, counseling referrals, information on experiences from other patients/caregivers, contact with specialist nursing teams and online support groups. While online support groups were only explicitly mentioned by 1 participant in the first round, over 70% of the participants in the second round agreed that PAGs provide online support groups. Main caregiver challenges identified were providing emotional support to their loved one while needing it themselves (*n* = 22/26, 85%), dealing with anticipatory grief (*n* = 21/26, 81%), managing uncertainty (*n* = 20/26, 77%), and having to research GBM to be able to access support for patient needs (*n* = 19/26, 73%), as visualized in Figure 4a.

### Patient and Caregiver Quality of Life

Several factors may negatively impact patient and caregiver quality of life. For patient quality of life, participants reached consensus that these factors include treatment side effects (*n* = 23/26, 88%), loss of independence (*n* = 22/26, 85%), grief over loss of future (*n* = 22/26, 85%), uncertainty/fear (*n* = 21/26, 81%), inability to do formerly loved activities (*n* = 21/26, 81%), cognitive/speech/sight/mobility losses (*n* = 20/26, 77%), and anxiety/depression (*n* = 19/26, 73%). For caregivers, participants agreed that uncertainty/fear (*n* = 23/26, 88%), emotional impact of their loved one’s cognitive decline (*n* = 21/26, 81%), psychological toll of communicating with family/friends (*n* = 21/26, 81%), anticipatory grief (*n* = 20/26, 77%), and stress associated with helping their loved one cope with side effects (*n* = 19/26, 73%) negatively affect their quality of life. A caregiver reported that they were “paying privately for counseling” because they were very traumatized by their experience of GBM care and management for their son. “We will never recover from this,” they stated. The results for this domain are visualized in panels (b and c) in Figure 4.

### Measures to Ease the Burden of Glioblastoma

**Figure 5.**
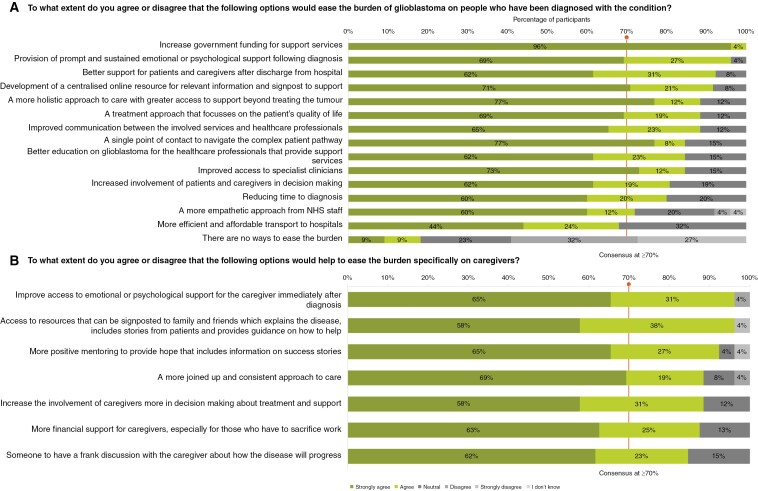
Participant responses ranking measures that may help ease the burden on (a) people with glioblastoma and (b) their caregivers. Consensus was defined as ≥70% agreement between participants. Responses were ranked according to the level of agreement as follows: “Strongly agree”; “Agree”; “Neutral”; “Disagree”; “Strongly disagree”; “I don’t know.” All 26 participants responded to both questions but 4 of them skipped some elements of the question shown in panel (a), and 2 skipped some elements of the question shown in panel (b). Abbreviation: NHS (National Health Service).

On easing the patient burden of GBM, participants reached a consensus on 13 factors that would help (Figure 5a). These factors are increasing government funding for brain tumor research (*n* = 26/26, 100%), prompt psychological support (*n* = 25/26, 96%), better postdischarge support (*n* = 24/26, 92%), a centralized online information hub (*n* = 22/24, 92%), more holistic care beyond treatment (*n* = 23/26, 88%), patient-focused treatment (*n* = 23/26, 88%), improved communication between services (*n* = 23/26, 88%), specialist access (*n* = 22/26, 85%), education for professionals (*n* = 22/26, 85%), patient/caregiver involvement in decisions (*n* = 21/26, 81%), faster diagnosis (*n* = 20/25, 80%), and a more empathetic approach from NHS staff (*n* = 18/25, 72%).

Participants also reached a consensus on all 7 factors identified in the questionnaire that may ease the burden of GBM on the caregivers (Figure 5b). The highest level of consensus among participants was on 2 of the 7 factors which include improving access to emotional or psychological support for the caregiver immediately after diagnosis (*n* = 25/26, 96%) and access to centralized resources that can be signposted to family and friends which explain the disease, include stories from patients and provide guidance on how to help (*n* = 25/26, 96%). Other factors identified were more positive mentoring for caregivers to provide hope that includes information on success stories (*n* = 24/26, 92%), more financial support for caregivers, especially for those who have to sacrifice work to look after their loved one (*n* = 21/24, 88%), increasing the involvement of caregivers in decision making about treatment and support (*n* = 23/26, 88%), a more coordinated and consistent approach to care (*n* = 23/26, 88%), and someone to have a frank discussion with the caregiver about how the disease will progress (*n* = 22/26, 85%). The results for this domain are visualized in Figure 5.

### Access to Clinical Trials and New Treatments

**Figure 6.**
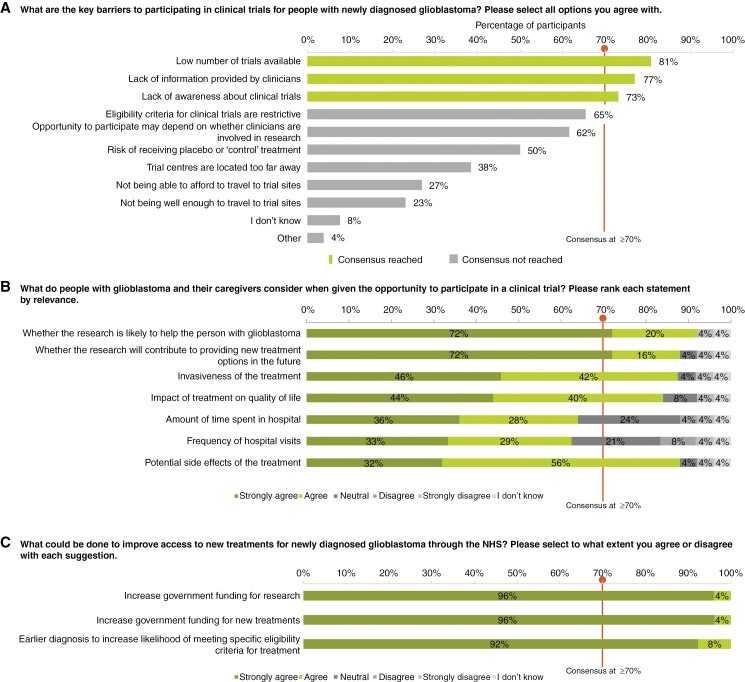
Participant responses on the key (a) barriers and (b) considerations in participating in clinical trials and (c) measures to improve access to new treatments. Consensus was defined as≥70% agreement between participants. In panels (b) and (c) responses were ranked according to level of agreement as follows: “Strongly agree”; “Agree”; “Neutral”; “Disagree”; “Strongly disagree”; “I don’t know.” All 26 participants responded to questions in panels (a) and (c), but 1 participant skipped some elements of the question in panel (c). A total of 25 of the 26 participants responded to the question in panel (b), and 1 of those participants skipped parts of that question. Abbreviation: NHS (National Health Service).

There are several barriers for people with newly diagnosed GBM to participate in clinical trials. Participants reached a consensus that low trial availability (*n* = 21/26, 81%), lack of information from clinicians about clinical trials (*n* = 20/26, 77%), and lack of awareness among patients and caregivers about accessible clinical trials (*n* = 19/26, 73%) may be the key barriers. Over 70% agreed that when deciding whether to participate in a clinical trial, they consider trial benefits for the patient (*n* = 23/25, 92%) and future patients (*n* = 22/25, 88%), potential side effects (*n* = 22/25, 88%), invasiveness (*n* = 21/24, 88%), and impact on quality of life (*n* = 21/25, 84%) (Figure 6a). A participant reported that they “would certainly consider the opportunity [of participating in a clinical trial] to be of some benefit to someone in the future” (Figure 6b). No consensus was reached on how accessible clinical trials are for people with newly diagnosed GBM, with the most common response (*n* = 9/26, 35%) being “I don’t know.”

There was 100% consensus—(*n* = 26/26) participants “strongly agreed” or “agreed”—that increased government funding for research and new treatments would improve access to new treatments within the NHS. All participants (*n* = 26/26, 100%) also agreed that earlier diagnosis would help boost access to new treatments by enabling newly diagnosed patients to meet trial eligibility criteria for particular studies appropriate in the newly diagnosed setting (Figure 6c).

### Impact of COVID-19

The participants agreed that, in the United Kingdom, the COVID-19 pandemic had a negative impact on the diagnosis, treatment, and support of people with newly diagnosed GBM and their caregivers. Out of 26 total participants, 73% (*n* = 19/26) agreed that the pandemic had a negative impact on this patient population. One participant reported that COVID-19 had slowed down the treatment pathway for their father and that although his tumor was detected, it was not sufficiently monitored during the pandemic until he had his first seizure 6 months after detection. As a result, it had grown aggressively by December of the same year, when he had his first seizure. Another participant reported that they were not able to be with their son at his NHS appointments and that they had decreased access to his consultant due to the pandemic, which made it difficult to make inquiries and get information. A similar account was reported by another caregiver. “When our son was diagnosed with a brain tumor my husband wasn’t even allowed in the hospital to visit us,” they stated.

## Discussion

Our study utilized 2 rounds of the Delphi panel to identify key common issues that newly diagnosed GBM patients and their caregivers face in the United Kingdom and the measures they think will mitigate these issues, in their journey from diagnosis to treatment and beyond. Participants agreed that A&E visits are the most common route to a GBM diagnosis, despite the latest National Cancer Patient Experience Survey (NCPES) results published in 2022, showing that 60% of brain cancer patients in England have spoken to their GP at least twice about their symptoms before being diagnosed.^15^ This suggests brain cancer patients endure worsening, nonspecific symptoms, and repeat primary care consultations before urgent hospital admission.^16,17^

Our study found that information overload about the disease subsequent lifestyle changes and emotional distress at the time of diagnosis were the main challenges patients and caregivers face on receiving a GBM diagnosis. This reinforces the findings of a previous longitudinal study of 15 patients over a period of 2 years by Sutton et al. In this series of interviews exploring the subjective well-being of patients with GBM, emotional distress and identity crisis on receiving their diagnosis were among the common themes.^9^ There is a clear need for improving the diagnostic pathway for GBM patients in the United Kingdom, a more holistic approach to care is needed. Healthcare professionals should also expect and be prepared, through education and communication processes, to appropriately address the overwhelm and difficulty of processing complex information that patients and caregivers experience during this time.^9,18^

The limitations of currently available NHS treatments for GBM identified by participants in our study have also previously been reported to be similar in other parts of the world. In a narrative review of key clinical trials and research studies in the field of newly diagnosed GBM, Theeler et al. reported the main limitations of current GBM treatments. The drawbacks included poor efficacy, significant side effects, lack of personalization, and invasiveness of surgical resection,^19^ of which are in line with our findings, despite the 8-year gap between our study and the referenced research.

Additionally, our study also reported on challenges around care coordination and communication with and among healthcare professionals indicating a gap in the provision of practical support. The NCPES results further explained such gaps reporting that, in England, 25.3% of brain cancer patients do not receive information about how to get financial help or benefits they might be entitled to and that 41.0% are not informed that they can get a second opinion or further advice from a healthcare professional before making decisions.^15^ McKinnon et al. explained in a clinical update that GBM patients and their caregivers often undergo a rollercoaster of emotions, experiencing worry and distress as they navigate their individual journeys through the challenges of GBM. Throughout the entire continuum of care, it is crucial for healthcare professionals to recognize and understand these common experiences to be able to offer appropriate support and engage in sensitive and effective communication, from the moment of diagnosis to providing bereavement support to the caregiver.^18^

Several studies have also found that GBM caregiver distress and psychological morbidity often exceeds that of patients.^4,20,21^ Boele et al. reported in a longitudinal study of 88 patient-caregiver dyads that 77% of caregivers and 69% of GBM patients experience high levels of distress, anxiety, depression, and reduced quality of life after diagnosis and during treatment.^4^ Adopting early palliative care with MDT coordination can enhance the quality of life for GBM patients and caregivers, in line with various guidelines, including the shared decision-making guideline by the National Institute for Health and Care Excellence (NICE). These guidelines recommend greater patient/caregiver involvement, centralized care navigation, accessible information resources, and specialist access.^4,18,22,23^ Our findings lend support from the patient and caregiver perspective for implementing these types of recommendations. In addition, the development and implementation of a core outcome set and patient-reportable outcomes for GBM trials, as proposed by the COBra project, could enhance the relevance and comparability of research evidence from clinical trials and subsequently, improve the quality of life and satisfaction of patients and caregivers.^24^

Barriers also exist which prevent patient access to clinical trials, such as lack of awareness among patients and caregivers, and lack of information provided by healthcare professionals as reported by patients and caregivers in our study. Our findings are in line with an All-Party Parliamentary Group on Brain Tumours report, which identified patient-level barriers in the United Kingdom.^25^ The report explained that few patients believe that healthcare professionals give sufficient information about opportunities to participate in clinical research and that clinical trials are often inaccessible to brain tumor patients who frequently have physical disabilities and poor health that make it difficult for them to travel long distances to the trial site.^25^ There have also been reports of failure of clinicians to discuss clinical trials with patients.^26^ According to the NCPES results published in 2022, 48.5% of brain cancer patients in England reported that they were not informed of cancer research opportunities they could have potentially participated in although they would have liked to.^15^ A recently published review by Bagley et al. provided further recommendations from the Society for Neuro-Oncology (SNO) Think Tank held in 2020, to improve accessibility to GBM clinical trials. These recommendations include relaxing eligibility criteria where possible, limiting randomization to control arms by using adaptive platform trial designs to increase patient willingness to participate, and setting up cooperative groups to jointly lead key GBM trials, facilitating accrual across multiple centers.^27^ There is a clear need for improving the awareness of opportunities to participate in clinical trials and empower patient and caregiver decision-making.^27,28^

Participants in our study reached a consensus that COVID-19 further worsened the mental health, anxiety, access to services, and quality of life of brain tumor patients and their caregivers. This is in line with the findings of a study by Voisin et al. based on a global survey developed by IBTA in conjunction with the SNO COVID-19 Task Force.^29^ The study surveyed 1989 brain tumor patients and caregivers from 33 countries and found that COVID-19 had a significant impact on their mental health and anxiety, with patients experiencing delays in treatments and appointments while caregivers felt isolated, stressed and unsupported.^29^ Another prospective national survey of neurosurgical units in the United Kingdom, by Price et al., performed between March 23 and April 24, 2020, during the peak of the first wave of the COVID-19 pandemic, provided further insight into the impact of COVID-19 on changes in initial management decisions for patients with brain tumors by MDTs. In this study, the data on 1221 new patients discussed at 80 MDT meetings revealed that 10.7% of patients (*n* = 131/1221) had a change in initial management due to COVID-19, with the majority (68%) not undergoing surgery and over half receiving no active treatment during the study period.^30^

However, it should be noted that, while a consensus was reached in our study, 27% of the participants did not “strongly agree” or “agree” that COVID-19 had a negative impact on the diagnosis, treatment, and support of people with newly diagnosed GBM and their caregivers. This may indicate that some participants had positive or neutral experiences during the pandemic, or that they did not perceive COVID-19 as a major factor affecting their care. This variation in responses may depend on additional factors like differences in personal circumstances, coping strategies, expectations and preferences of the participants, and when the participants were diagnosed. This variation in UK experiences has not previously been explored in currently available studies. There is also a need for more psychological support and education for GBM patients and their caregivers, as well as for health professionals who may face increased workload and stress.^18^

### Strengths and Limitations

This study had several strengths, including the diverse sample of patients and caregivers which enhanced representativeness and credibility. By recruiting participants from 4 different PAGs, perspectives were gathered from a variety of experiences with GBM. A high level of consensus was reached on 7 out of 9 domains explored in the Delphi panels, indicating strong agreement on key issues related to GBM in the United Kingdom. The structured Delphi panel methodology promoted honest, independent opinions from participants through controlled, anonymous communication and feedback. PAG representatives were involved in an initial preparatory roundtable discussion which informed the development of the Delphi panel questionnaires based on insights from GBM expert patient advocates.

However, as participants remained anonymous, there was a partial lack of continuity in the sample between the first and second rounds of the Delphi panel, meaning the same participants could not be ensured in both rounds. Maximum continuity is preferred in Delphi studies to maintain the full breadth of perspectives throughout the iterative rounds and to allow interpretable refinement of the group’s consensus. Disrupted continuity risks losing minority viewpoints and introducing inconsistencies in the perspective evolution. This lack of continuity, combined with the relatively small sample sizes in each round, may have impacted the diversity of opinions captured. Distribution through PAG channels also meant the number of participants could not be strictly controlled between rounds, though attempts were made to keep it consistent, and the questionnaires were distributed to the same group of individuals in each round. As the questionnaires were distributed to the same group of individuals affiliated with the 4 participating PAGs in each round, there was likely some overlap in the patient and contact lists across these advocacy groups. This potential overlap in the pools from which participants were drawn may have further limited the diversity of perspectives captured in the study.

Participants’ personal experiences with GBM varied, and the variance in knowledge of diagnostic processes and treatment complications could have introduced inadvertent biases. Moreover, participation in the study was voluntary based on responses to open invitations from the PAGs, which may have biased the sample toward those with more extreme experiences or motivation to express concerns and those from particular socio-economic backgrounds. Another key limitation was the lack of representation of patients and caregivers from non-English speaking backgrounds. These populations might face heightened barriers navigating the GBM diagnostic and treatment landscape compared to English-speaking patients in the UK, which restricts the generalizability of the findings to diverse socio-cultural contexts.

Overall, while some limitations existed, high consensus, and anonymity allowed honest opinions and this Delphi panel succeeded in capturing impactful insights into patient experiences with GBM in the United Kingdom. Our study also lays the groundwork for further investigations on this topic.

## Conclusion

The consensus achieved across multiple domains highlights key issues in the care of people with newly diagnosed GBM in the United Kingdom. Results of the Delphi panel indicate that the diagnosis pathway in the United Kingdom is inconsistent, as diagnosis can occur through diverse pathways for patients, with a significant proportion of GBM diagnoses occurring through an A&E visit. Receiving a GBM diagnosis is extremely challenging and is associated with a lack of emotional support and information overload. The Delphi panel also highlights that treatment pathways are not patient-centric or holistic and lack effective and minimally invasive treatment options. This issue, together with the aggressive nature of GBM and associated side effects with current treatments, severely affects the quality of life of patients and their caregivers. Patients and caregivers who participated in the Delphi panel experience challenges associated with the coordination of GBM care among MDTs in the United Kingdom. Moreover, the Delphi panel participants agreed that access to new treatments and clinical trials is limited due to lack of suitable trials, lack of information relay by clinicians and lack of awareness among patients. The COVID-19 pandemic imposed a further burden on patients and their caregivers. In addition, they also agreed that increased government funding for brain tumor research is required.

The patient advocacy organization community recognizes the severe burden of GBM on patients and caregivers and calls for increased funding for research in GBM and access to new treatments. The community advocates for a holistic approach to GBM treatment and management and improved support for and from healthcare professionals. This would include enhanced education and training specifically tailored for A&E specialists to better equip these clinicians to sensitively convey a new brain tumor diagnosis and make appropriate referrals.

## Supplementary material

Supplementary material is available online at *Neuro-Oncology Practice* (https://academic.oup.com/nop).

npae058_suppl_Supplementary_Table_S1
