# Supplementary material for: Challenges and opportunities in newly diagnosed glioblastoma in the United Kingdom: A Delphi panel
Source: Neurooncol Pract. 2024 Jun 22;11(6):740–52. doi: 10.1093/nop/npae058 (PMC11567735; doi:10.1093/nop/npae058)
Supplement: npae058_suppl_Supplementary_Table_S1 [file npae058_suppl_supplementary_table_s1.docx]

# Supplementary materials:

Participant demographics: Table S1

Delphi panel questionnaires:

First round questionnaire

Second round questionnaire

Participant demographics

Table S1. Demographic characteristics of participants in the first and second rounds of the Delphi panel

| **Demographic characteristics n (%)** | **First round (n=17)** | **Second round (n=26)** |
| --- | --- | --- |
| Patient living with GBM | 8 (47%) | 12 (46%) |
| Caregiver for someone with GBM | 9 (53%) | 13 (50%) |
| Patient representative | 0 (0%) | 1 (4%) |
| **Region within the UK** | | |
| London | 1 (6%) | 0 (0%) |
| Wales | 1 (6%) | 0 (0%) |
| Northern Ireland | 0 (0%) | 0 (0%) |
| North West of England | 4 (24%) | 2 (8%) |
| Yorkshire and The Humber | 2 (12%) | 2 (8%) |
| South East of England | 3 (18%) | 9 (35%) |
| East of England | 1 (6%) | 4 (15%) |
| East Midlands | 1 (6%) | 4 (15%) |
| West Midlands | 1 (6%) | 4 (15%) |
| South West of England | 3 (18%) | 0 (0%) |
| North East of England | 0 (0%) | 0 (0%) |
| Scotland | 0 (0%) | 1 (4%) |
| **Age** | | |
| 0 to <18 years old | 2 (12%) | 3 (12%) |
| 18 to 30 years old | 1 (6%) | 1 (4%) |
| 31 to 40 years old | 1 (6%) | 2 (8%) |
| 41 to 50 years old | 1 (6%) | 5 (19%) |
| 51 to 60 years old | 7 (41%) | 6 (23%) |
| 61 to 70 years old | 4 (24%) | 7 (27%) |
| 71 to 80 years old | 1 (6%) | 0 (0%) |
| >80 years old | 0 (0%) | 0 (0%) |

Abbreviations: GBM (glioblastoma multiforme); n (number of participants); UK (United Kingdom)

Glioblastoma Delphi study – first round questionnaire

Thank you for participating in this Delphi panel study on glioblastoma in the UK and for taking the time to provide your valuable input. The aim of this study is to reach consensus on the current treatment pathway for people with newly diagnosed glioblastoma, the associated challenges and unmet needs and opportunities that could help to ease the burden on them and on their caregivers.

- Prior to completing this questionnaire, please consider reading the provided Guideline Document for Participants with more details on the objectives, Delphi panel methodology, as well as what is expected from you and when each step will take place.
- Your participation in this Delphi panel study will be completely anonymous. No personal information will be collected that could identify you. The use of Microsoft Forms for the completion of this online questionnaire allows you to participate anonymously. All responses will be handled by a Novocure’s third party facilitator (Facilitator).
- The first-round questionnaire consists of 24 open-ended questions. The subsequent two rounds should largely consist of closed questions.
- Your answer should reflect what you believe to be the answer to the question according to your expert opinion as someone affected by glioblastoma, a caregiver or patient representative.
- Please skip questions that you feel do not apply to you.
- Following your completion of this questionnaire, the Facilitator will analyse all responses and develop a second questionnaire with closed questions. The second questionnaire is expected to be available for your completion in the week commencing 3^rd^ April 2023; a third questionnaire is expected to be available in the week commencing 5^th^ June 2023.

Participant information

1. Are you someone living with glioblastoma, a caregiver or patient representative?

Type of question: Polychotomous, one possible answer

| I am someone living with glioblastoma (Go to question iii) |
| --- |
| I am a caregiver for someone with glioblastoma |
| I am a patient representative (Go to question iii) |
| Other (please specify) (Go to question iii) |

1. If you are a caregiver, what is your relation to the person with glioblastoma?

Type of question: Polychotomous, one possible answer

| Close family member (e.g. parent, sibling, offspring, aunt/uncle, cousin, grandparent) |
| --- |
| Close friend |
| Employed carer |
| Other (please specify) |

1. If you are someone living with a brain tumour, what disease are you diagnosed with? If you are a caregiver for someone with a brain tumour, what disease does the person you care for has been diagnosed with? If you are a patient representative or researcher, what disease do you have expertise in?

Type of question: Polychotomous, one possible answer

| Glioblastoma |
| --- |
| Other brain tumour (please specify) |

1. In which region/country in the UK are you based?

Type of question: Polychotomous, one possible answer

| Scotland |
| --- |
| Wales |
| Northern Ireland |
| North West of England |
| North East of England |
| Yorkshire and The Humber |
| East Midlands |
| West Midlands |
| East of England |
| London |
| South East of England |
| South West of England |

1. If you are someone living with glioblastoma, what is your age? If you are a caregiver for someone with glioblastoma, what is the age of the person you provide care for?

Type of question: Polychotomous, one possible answer

| Not applicable (I am a patient representative or researcher) |
| --- |
| 0 to <18 years old |
| 18 to 30 years old |
| 31 to 40 years old |
| 41 to 50 years old |
| 51 to 60 years old |
| 61 to 70 years old |
| 71 to 80 years old |
| >80 years old |

Diagnosis of glioblastoma in the UK

1. How are people with glioblastoma diagnosed in the UK?

Type of question: Open

1. What are the challenges associated with receiving a diagnosis of glioblastoma?

Type of question: Open

1. How quickly do people receive support after they are diagnosed with glioblastoma?

Type of question: Open

NHS patient and treatment pathways

1. To what extent do people with newly diagnosed glioblastoma receive optimal care and treatment?

Type of question: Open

1. What are the challenges and unmet needs in the NHS patient pathway for people with glioblastoma in the NHS?

Type of question: Open

1. What do you identify as the limitations of treatments currently available for newly diagnosed glioblastoma in the NHS?

Type of question: Open

Integrated care and multidisciplinary teams (MDTs)

A multidisciplinary team (MDT) is a group of health and care staff who are members of different organisations and professions (e.g. GPs, social workers, nurses) that work together to provide integrated care and make decisions about how best to care and treat individual patients.

1. How integrated is the care and treatment people with glioblastoma receive? Please consider how different teams work closely with one another if there is an MDT involved.

Type of question: Open

1. What are the challenges of being provided with integrated care by MDTs for people with glioblastoma?

Type of question: Open

1. How could integrated care be improved for people with glioblastoma?

Type of question: Open

1. Do you think that the quality of care of people with newly diagnosed glioblastoma differs depending on where they live?

Type of question: Open

Support beyond treatment

1. What support within the NHS is provided to people with newly diagnosed glioblastoma aside from treatment?

Type of question: Open

1. How could these support services be improved?

Type of question: Open

1. What are some of the main barriers that caregivers experience when looking after someone with glioblastoma?

Type of question: Open

1. What support do patient advocacy groups provide to patients with newly diagnosed glioblastoma?

Type of question: Open

Patient and caregiver quality of life

1. In what ways does glioblastoma affect the quality of life of people with the condition?

Type of question: Open

1. In what ways does glioblastoma affect the quality of life of caregivers for people with the condition?

Type of question: Open

Access to new treatments on the NHS

1. What do you think are the barriers to access to new treatments for newly diagnosed glioblastoma in the UK?

Type of question: Open

1. What could be done to improve access to new treatments for newly diagnosed glioblastoma through the NHS?

Type of question: Open

Access to clinical trials

1. How accessible are clinical trials for people with newly diagnosed glioblastoma?

Type of question: Open

1. What are the barriers to people with newly diagnosed glioblastoma of participating in clinical trials?

Type of question: Open

1. What are the considerations of people with glioblastoma and their caregivers when given the opportunity to participate in a clinical trial?

Type of question: Open

Measures to ease the burden of glioblastoma

1. What could be done to help ease the burden of glioblastoma on people who have been diagnosed with the condition?

Type of question: Open

1. What could be done to help ease the burden on caregivers looking after someone with newly diagnosed glioblastoma?

Type of question: Open

Impact of COVID-19 on glioblastoma

1. What has been the impact of the COVID-19 pandemic on the diagnosis, treatment and support of people with newly diagnosed glioblastoma?

Type of question: Open

Delphi panel questionnaires: Second-round questionnaire

**Glioblastoma Delphi study – second round questionnaire**

Thank you for participating in this Delphi panel study on glioblastoma in the UK and for taking the time to provide your valuable input. The aim of this study is to reach consensus on the current treatment pathway for people with newly diagnosed glioblastoma, the associated challenges and unmet needs and opportunities that could help to ease the burden on them and on their caregivers.

- The second-round questionnaire consists of 21 closed, multiple-choice questions preceded by 6 multiple-choice questions about the participant. The third and final round should also consist of closed questions.
- The deadline to complete this questionnaire is **Sunday 23^rd^ April 2023 at 23:59**.
- Your participation in this Delphi panel study will be completely anonymous. No personal information will be collected that could identify you. The use of Microsoft Forms for the completion of this online questionnaire allows you to participate anonymously. All responses will be handled by Novocure’s third party facilitator (“Facilitator”).
- Your answers should reflect what you believe to be the answer to the question, according to your expert opinion as someone affected by glioblastoma, a caregiver, or a patient representative.
- Please read the questions carefully and please skip any questions that are not applicable to you.
- Following your completion of this questionnaire, the Facilitator will analyse all responses and develop a third questionnaire if consensus is not reached in the second round. The third questionnaire is expected to be available for your completion in the week commencing 5^th^ June 2023.

Participant information (please answer these questions again, even if you participated in the first round)

1. Did you participate in the first-round questionnaire?

| Yes |
| --- |
| No |

1. Are you someone living with glioblastoma, a caregiver, or patient representative?

| I am someone living with glioblastoma (Go to question 4) |
| --- |
| I am a caregiver for someone with glioblastoma |
| I am a patient representative (Go to question 4) |
| Other (please specify) (Go to question 4) |

1. If you are a caregiver, what is your relation to the person with glioblastoma?

| Close family member (e.g. parent, sibling, offspring, aunt/uncle, cousin, grandparent) |
| --- |
| Close friend |
| Employed carer |
| Other (please specify) |

1. If you are someone living with a brain tumour, what disease are you diagnosed with? If you are a caregiver for someone with a brain tumour, what disease does the person you care for has been diagnosed with? If you are a patient representative or researcher, what disease do you have expertise in?

| Glioblastoma |
| --- |
| Other brain tumour (please specify) |

1. In which region/country in the UK are you based?

| Scotland |
| --- |
| Wales |
| Northern Ireland |
| North West of England |
| North East of England |
| Yorkshire and The Humber |
| East Midlands |
| West Midlands |
| East of England |
| London |
| South East of England |
| South West of England |

1. If you are someone living with glioblastoma, what is your age? If you are a caregiver for someone with glioblastoma, what is the age of the person you provide care for?

| Not applicable (I am a patient representative or researcher) |
| --- |
| 0 to <18 years old |
| 18 to 30 years old |
| 31 to 40 years old |
| 41 to 50 years old |
| 51 to 60 years old |
| 61 to 70 years old |
| 71 to 80 years old |
| >80 years old |

Diagnosis of glioblastoma in the UK

1. What are the most common routes to a glioblastoma diagnosis? Please select all options that apply.

| Visit to a GP |
| --- |
| Visit to a GP followed by an appointment with a specialist |
| Visit to an optician |
| Visit to a paediatrician |
| Visit to A&E |
| Other (please specify) |

1. What are the challenges associated with receiving a diagnosis of glioblastoma? Please select all options you agree with.

| Difficulty securing a GP appointment in the first instance |
| --- |
| Delay in diagnosis as symptoms are not specific to glioblastoma |
| Emotional or psychological challenges: dealing with shock, lack of hope, fear of mortality and/or uncertainty about the future |
| Lots of information to take in |
| Administrative burden and medical appointments |
| Difficulty finding support groups |
| Lack of emotional or psychological support provided by the NHS |
| Lack of educational materials provided by the NHS |
| Lack of a single place to get information |
| Requirement to proactively research and find support |
| Financial concerns associated with inability to work |
| Other (please specify) |

NHS patient and treatment pathways

1. How quickly do people receive the following types of support after they are diagnosed with glioblastoma? Please select an option for each type of support.

|  | Within 1 week | Within 2 weeks | Within 3 weeks | Within 4 weeks | Longer than 4 weeks | Generally not available | I don’t know |
| --- | --- | --- | --- | --- | --- | --- | --- |
| Follow-up medical appointment |  |  |  |  |  |  |  |
| Start of treatment |  |  |  |  |  |  |  |
| Emotional or psychological support |  |  |  |  |  |  |  |
| Financial support |  |  |  |  |  |  |  |

1. How would you rank the following aspects of care for people with newly diagnosed glioblastoma?

|  | Very good | Good | Fair | Poor | Very poor | I don’t know |
| --- | --- | --- | --- | --- | --- | --- |
| Quality of care received from GPs |  |  |  |  |  |  |
| Knowledge/expertise of GPs in glioblastoma |  |  |  |  |  |  |
| Quality of care received from specialist clinicians |  |  |  |  |  |  |
| Knowledge/expertise of specialist clinicians in glioblastoma |  |  |  |  |  |  |
| Quality of communication from clinicians |  |  |  |  |  |  |
| Treatment experience |  |  |  |  |  |  |

1. What are the challenges and unmet needs in the NHS patient pathway for people with newly diagnosed glioblastoma? Please rank each option in terms of relevance.

|  | Highly relevant | Relevant | Neutral | Less relevant | Not relevant | I don’t know |
| --- | --- | --- | --- | --- | --- | --- |
| Lack of effective treatment options |  |  |  |  |  |  |
| Treatment options have not progressed for decades |  |  |  |  |  |  |
| Access to care and treatment is delayed or inadequate |  |  |  |  |  |  |
| Communication between healthcare professionals is poor |  |  |  |  |  |  |
| Lack of personalised therapeutic approach |  |  |  |  |  |  |
| Short-term approach without proper consideration to patients’ quality of life |  |  |  |  |  |  |
| Need for a more holistic approach to care, including areas of support beyond treating the tumour |  |  |  |  |  |  |
| Lack of support if treatment fails |  |  |  |  |  |  |
| Lack of hope from healthcare professionals |  |  |  |  |  |  |

1. What do you identify as the limitations of treatments currently available for newly diagnosed glioblastoma in the NHS? Please rank each limitation by relevance.

|  | Highly relevant | Relevant | Neutral | Less relevant | Not relevant | I don’t know |
| --- | --- | --- | --- | --- | --- | --- |
| Not curative |  |  |  |  |  |  |
| Poor efficacy |  |  |  |  |  |  |
| Too invasive |  |  |  |  |  |  |
| Associated with significant side effects |  |  |  |  |  |  |
| Not personalised |  |  |  |  |  |  |

Integrated care and multidisciplinary teams (MDTs)

A multidisciplinary team (MDT) is a group of health and care staff who are members of different organisations and professions (e.g. GPs, social workers, nurses) that work together to provide integrated care and make decisions about how best to care and treat individual patients.

1. To what extent do you feel that care for people with glioblastoma is joined up? Please select to what extent you agree or disagree with each statement.

|  | Strongly agree | Agree | Neutral | Disagree | Strongly disagree | I don’t know |
| --- | --- | --- | --- | --- | --- | --- |
| MDTs work for the benefit of the patient. |  |  |  |  |  |  |
| MDTs are well joined up. |  |  |  |  |  |  |
| Transitions between services are well managed. |  |  |  |  |  |  |
| Communication between individuals and services is good. |  |  |  |  |  |  |

1. What are the challenges of being provided with care by MDTs for people with glioblastoma? Please rank each challenge by relevance.

|  | Highly relevant | Relevant | Neutral | Less relevant | Not relevant | I don’t know |
| --- | --- | --- | --- | --- | --- | --- |
| Difficulty retaining and comprehending information |  |  |  |  |  |  |
| Lack of timely communication between healthcare professionals |  |  |  |  |  |  |
| Difficulty in reaching and communicating with healthcare professionals |  |  |  |  |  |  |
| Difficulty knowing who and how to ask for necessary support |  |  |  |  |  |  |
| Some healthcare professionals lack understanding of the needs of people with glioblastoma |  |  |  |  |  |  |
| There are no challenges associated with being provided with integrated care by MDTs |  |  |  |  |  |  |

1. To what extent do you agree or disagree that the following aspects of care differ depending on where people with glioblastoma live in the UK?

|  | Strongly agree | Agree | Neutral | Disagree | Strongly disagree | I don’t know |
| --- | --- | --- | --- | --- | --- | --- |
| Quality of care |  |  |  |  |  |  |
| Access to support beyond treating the tumour |  |  |  |  |  |  |
| Expertise of clinicians in glioblastoma |  |  |  |  |  |  |

Support beyond treatment

1. What support within the NHS is provided to people with newly diagnosed glioblastoma aside from treatment? Please select all options you agree with.

| Nursing support |
| --- |
| Speech therapy |
| Occupational therapy |
| Physiotherapy |
| Emotional or psychological support |
| Dietary support |
| Neuropsychologist support |
| Financial support |
| Signposting to charities and patient advocacy groups (PAGs) |
| A hotline phone number |
| Mobility aids/equipment |
| None |
| Other (please specify) |

1. What are the main challenges that caregivers experience when looking after someone with glioblastoma? Please select all options you agree with.

| Difficulty managing increased day-to-day responsibilities |
| --- |
| Providing emotional or psychological support whilst feeling that they themselves need this type of support |
| Needing to research glioblastoma to be able to request support to address the patient’s needs |
| Lack of NHS support for caregivers |
| Dealing with anticipatory grief |
| Lack of quality time to spend with the person they are caring for |
| Anxiety and fear associated with helping the person they are caring to cope with symptoms (e.g. seizures) |
| Managing the uncertainty of the ever-changing progression of the disease |
| Financial challenges associated with travel costs and loss of income |
| There are no barriers |
| I don’t know |
| Other (please specify) |

1. What support do Patient Advocacy Groups (PAGs) or charities provide for people with newly diagnosed glioblastoma? Please select all options that apply.

| Online support groups |
| --- |
| Financial advice |
| Community meet-ups |
| Befriending phone services |
| Campaigning to address issues |
| Information and guidance |
| Signposting to relevant support and services |
| Kindness and empathy |
| I don’t know |
| Other (please specify) |

Patient and caregiver quality of life

1. What are the main factors that negatively affect the quality of life of people with glioblastoma? Please select all options you agree with.

| Lack of independence |
| --- |
| Lack of ability to work |
| Uncertainty/fear of the unknown |
| Available treatments have undesired side effects |
| Available treatments are invasive and/or may require long hospital stays |
| Frequency of hospital visits |
| Loss of cognitive function, speech, sight and/or mobility |
| Depression and anxiety |
| No longer being able to do the things you once loved |
| Lack of optimism |
| Feeling of isolation |
| Grieving over loss of future |
| None |
| I don’t know |
| Other (please specify) |

1. What are the main factors that negatively affect the quality of life of caregivers. Please select all options you agree with.

| Financial difficulties |
| --- |
| Uncertainty/fear of the unknown |
| Emotional or psychological impact of cognitive decline caused by glioblastoma |
| Loss of independence |
| Anticipatory grief |
| Stress associated with helping the patient cope with the side effects of treatment |
| Emotional or psychological toll associated with communicating with friends and family |
| Managing increased day-to-day responsibilities |
| Difficulty ensuring the patient’s care needs are met |
| Change of identity from being a family member to becoming a full-time caregiver |
| I don’t know |
| Other (please specify) |

Measures to ease the burden of glioblastoma

1. To what extent do you agree or disagree that the following options would ease the burden of glioblastoma on people who have been diagnosed with the condition?

|  | Strongly agree | Agree | Neutral | Disagree | Strongly disagree |
| --- | --- | --- | --- | --- | --- |
| Reducing time to diagnosis |  |  |  |  |  |
| Development of a centralised online resource for relevant information and signpost to support |  |  |  |  |  |
| A single point of contact to navigate the complex patient pathway |  |  |  |  |  |
| A more holistic approach to care with greater access to support beyond treating the tumour |  |  |  |  |  |
| Provision of prompt and sustained emotional or psychological support following diagnosis |  |  |  |  |  |
| A treatment approach that focusses on the patient’s quality of life |  |  |  |  |  |
| Better support for patients and caregivers after discharge from hospital |  |  |  |  |  |
| Increased involvement of patients and caregivers in decision making |  |  |  |  |  |
| Improved access to specialist clinicians |  |  |  |  |  |
| Improved communication between the involved services and healthcare professionals |  |  |  |  |  |
| Better education on glioblastoma for the healthcare professionals that provide support services |  |  |  |  |  |
| A more empathetic approach from NHS staff |  |  |  |  |  |
| Increase government funding for support services |  |  |  |  |  |
| More efficient and affordable transport to hospitals |  |  |  |  |  |
| There are no ways to ease the burden |  |  |  |  |  |

1. To what extent do you agree or disagree that the following options would help to ease the burden specifically on caregivers?

|  | Strongly agree | Agree | Neutral | Disagree | Strongly disagree |
| --- | --- | --- | --- | --- | --- |
| Improve access to emotional or psychological support for the caregiver immediately after diagnosis |  |  |  |  |  |
| Someone to have a frank discussion with the caregiver about how the disease will progress |  |  |  |  |  |
| Access to resources that can be signposted to family and friends which explains the disease, includes stories from patients and provides guidance on how to help |  |  |  |  |  |
| A more joined up and consistent approach to care |  |  |  |  |  |
| More positive mentoring to provide hope that includes information on success stories |  |  |  |  |  |
| More financial support for caregivers, especially for those who have to sacrifice work |  |  |  |  |  |
| Increase the involvement of caregivers more in decision making about treatment and support |  |  |  |  |  |

Access to new treatments on the NHS

1. What could be done to improve access to new treatments for newly diagnosed glioblastoma through the NHS? Please select to what extent you agree or disagree with each suggestion.

|  | Strongly agree | Agree | Neutral | Disagree | Strongly disagree | I don’t know |
| --- | --- | --- | --- | --- | --- | --- |
| Increase government funding for research |  |  |  |  |  |  |
| Increase government funding for new treatments |  |  |  |  |  |  |
| Earlier diagnosis to increase likelihood of meeting specific eligibility criteria for treatment |  |  |  |  |  |  |

Access to clinical trials

1. How accessible are clinical trials for people with newly diagnosed glioblastoma?

| Highly accessible |
| --- |
| Somewhat accessible |
| Neutral |
| Somewhat inaccessible |
| Highly inaccessible |
| I don’t know |

1. What are the key barriers to participating in clinical trials for people with newly diagnosed glioblastoma? Please select all options you agree with.

| Lack of awareness about clinical trials |
| --- |
| Lack of information provided by clinicians |
| Opportunity to participate may depend on whether clinicians are involved in research |
| Trial centres are located too far away |
| Not being able to afford to travel to trial sites |
| Not being well enough to travel to trial sites |
| Low number of trials available |
| Risk of receiving placebo or ‘control’ treatment |
| Eligibility criteria for clinical trials are restrictive |
| I don’t know |
| Other (please specify) |

1. What do people with glioblastoma and their caregivers consider when given the opportunity to participate in a clinical trial? Please rank each statement by relevance.

|  | Highly relevant | Relevant | Neutral | Less relevant | Not relevant | I don’t know |
| --- | --- | --- | --- | --- | --- | --- |
| Potential side effects of the treatment |  |  |  |  |  |  |
| Impact of treatment on quality of life |  |  |  |  |  |  |
| Invasiveness of the treatment |  |  |  |  |  |  |
| Amount of time spent in hospital |  |  |  |  |  |  |
| Frequency of hospital visits |  |  |  |  |  |  |
| Whether the research is likely to help the person with glioblastoma |  |  |  |  |  |  |
| Whether the research will contribute to providing new treatment options in the future |  |  |  |  |  |  |

Impact of COVID-19 on glioblastoma

1. To what extent do you agree that the COVID-19 pandemic had a negative impact on the diagnosis, treatment and support of people with newly diagnosed glioblastoma and their caregivers?

| Strongly agree |
| --- |
| Agree |
| Neutral |
| Disagree |
| Strongly disagree |
| I don’t know |
